# Lipid and Glucose Profile across Different Mental Disorders

**DOI:** 10.3390/jcm13092499

**Published:** 2024-04-24

**Authors:** Derar H. Abdel-Qader, Abdullah Albassam, Esra’ Taybeh, Nadia Al Mazrouei, Sara Murad Albarkat Meer, Khalid Awad Al-Kubaisi, Rana Ibrahim, Asim Ahmed Elnour, Osama Mohamed Ibrahim, Salah AbuRuz

**Affiliations:** 1 Faculty of Pharmacy & Medical Sciences, University of Petra, Amman 11196, Jordan; d.balawi@igec.com.au; 2 Department of Pharmacy Practice, Faculty of Pharmacy, Kuwait University, Kuwait 12037, Kuwait; albassam@hsc.edu.kw; 3 Department of Applied Pharmaceutical Sciences, School of Pharmacy, Isra University, Amman 11622, Jordan; esra.taybeh@iu.edu.jo; 4 Department of Pharmacy Practice and Pharmacotherapeutics, College of Pharmacy, University of Sharjah, Sharjah 27272, United Arab Emirates; nalmazrouei@sharjah.ac.ae (N.A.M.); smeer@sharjah.ac.ae (S.M.A.M.); kalkubaissi@sharjah.ac.ae (K.A.A.-K.); ribrahim@sharjah.ac.ae (R.I.); 5 College of Pharmacy, Al Ain University, Abu Dhabi 112612, United Arab Emirates; asim.ahmed@aau.ac.ae; 6 AAU Health and Biomedical Research Center, Al Ain University, Abu Dhabi 112612, United Arab Emirates; 7 Department of Clinical Pharmacy, School of Pharmacy, New Giza University, Giza 3296121, Egypt; osama.hussein@ngu.edu.eg; 8 Department of Pharmacology and Therapeutics, College of Medicine and Health Sciences, The United Arab Emirates University, Al Ain 15551, United Arab Emirates

**Keywords:** schizophrenia, unipolar depression, bipolar disorder, bipolar mania, bipolar depression, lipid and glucose levels

## Abstract

**Objectives**: Schizophrenia, unipolar depression, bipolar disorder, bipolar mania, and bipolar depression are a few of the severe psychiatric diseases that affect millions of individuals and their overall life quality. This study aimed to look at differences in TGA, TC, HDL, LDL, and FPG levels in people who were going through acute episodes of listed diseases. **Materials and methods**: A cross-sectional prospective study was carried out in Jordan between January and November of 2023, involving all patients with the aforementioned diseases who attended three psychiatric clinics. This study encompassed results from 1187 patients (women N = 675, 56.87%) who were classified into the following ranges: <25, 25–45, 45–65, and >65. **Results**: The average level of LDL was the highest in bipolar depression (112.442 ± 36.178 mg/dL) and the lowest in bipolar mania (111.25 ± 33.14 mg/dL). The average level of HDL was the highest in schizophrenia (58.755 ± 16.198 mg/dL) and the lowest in bipolar depression (45.584 ± 12.128 mg/dL). Both average levels of TC and TGA were the highest in patients with bipolar depression (188.403 ± 37.396 mg/dL and 149.685 ± 96.951 mg/dL, respectively) and the lowest in bipolar mania (164.790 ± 40.488 mg/dL and 100.679 ± 54.337 mg/dL, respectively). The average level of FPG was the highest in unipolar depression (94.00 ± 21.453 mg/dL) and the lowest in bipolar mania (89.492 ± 14.700 mg/dL). **Conclusions**: The results confirmed that lipid and glucose abnormalities were more common in people with schizophrenia and mood disorders (unipolar and bipolar).

## 1. Introduction

Mental health is essential for each individual, everywhere [1]. However, it continues to be a low priority on the national and international agendas of funders and policymakers around the world [2]. The underprioritization of mental health and well-being has to be addressed.

The World Health Organization (WHO) estimates that one in four people worldwide suffer from mental illnesses, which greatly increases the burden of illness worldwide [3]. The mental health disorders schizophrenia, unipolar depression, bipolar disorder, bipolar depression, and bipolar mania are among the severe forms of psychiatric disorders that affect millions of people and their general well-being [4]. 

Moreover, these disorders are highly prevalent and have unique signs and pathways; however, emerging evidence suggests possible associations between mental health and physical health. Also, individuals with these disorders have a higher risk of dying from a variety of causes, including a higher chance of cardiovascular events, such as myocardial infarction, stroke, and sudden cardiac arrest [5]. The complex interplay between psychological and physical aspects raises critical questions about the overall health impacts of individuals with severe psychiatric conditions. These physical aspects, lipids, glucose, blood levels have drawn interest as possible indicators that go beyond mental symptomatology. 

Fasting plasma glucose (FPG) and lipid profiles, which include total cholesterol (TC), triglycerides (TGA), low-density lipoprotein (LDL), and high-density lipoprotein (HDL), are important markers of metabolic health. Disturbances in these indicators have been associated with wider metabolic dysregulation as well as cardiovascular illnesses [4].

Recent research [6] suggested a high prevalence of metabolic dysfunctions and mental health illness in patients. Understanding the link between mental health illness and metabolic dysregulation may provide important new information about the overall health profile of those with severe mental illness, especially since there is no previous investigation of this link conducted in Jordan. This research aimed to contribute to the continued advancement of knowledge in this field by conducting a systematic investigation of TGA, TC, HDL, LDL, and FPG levels in individuals with schizophrenia, unipolar depression, bipolar depression, bipolar disorder, and bipolar mania.

Although a previous study [4] compared differences in metabolic parameters between individuals suffering from severe forms of psychiatric disorders, it was a retrospective study. However, this study was a prospective study with fewer potential sources of bias and confounding than any retrospective study. 

There are important ramifications for general healthcare practice and mental health from this research. Comprehending the metabolic consequences of mental illnesses can aid medical professionals in creating comprehensive treatment plans that tackle psychological and physiological well-being. The results may also help develop preventative measures meant to lower the risk of metabolic and cardiovascular problems in those suffering from serious mental diseases.

## 2. Materials and Methods

### 2.1. Study Design and Data Collection

A prospective cross-sectional study was conducted in Jordan employing all patients with the stated disorders who visited three psychiatric clinics between January and November of 2023. A team of three consultant psychiatrists meticulously identified potential participants through clinic records, targeting those diagnosed with schizophrenia, unipolar depression, bipolar disorder, bipolar depression, or bipolar mania based on the DSM-IV explicit disorder criteria. Patients who refused the follow-up assessment were excluded from this study. Collaborating closely with clinic staff, we employed a systematic approach to invite eligible patients to take part in our study during their routine visits. Prior to inclusion, a detailed explanation of this study’s purpose and procedures was provided, ensuring a thorough understanding before obtaining written informed consent. This proactive recruitment strategy ensured a diverse and representative sample of the patient population. Participants in this study provided written, informed permission, and the Research Ethics Committee authorized this study (O/2/2/2023). 

Upon consenting to participate, patients were instructed to fast from midnight before their scheduled assessment. Blood samples were collected between 8 A.M. and 12 P.M., following strict adherence to fasting protocols. The laboratory analysis was conducted using standardized and calibrated equipment to ensure accuracy and reliability. We measured levels of TGA, TC, HDL, LDL, and FPG using high-precision assays. To guarantee the integrity of the data, the principal investigator meticulously collected, and another investigator double checked these lab results. This rigorous process was crucial in obtaining reliable and valid data for our study. This study comprised 1187 patients from various age groups.

### 2.2. Statistical Analysis

The statistical analysis was conducted using SPSS version 25. A one-way ANOVA test was used to assess the statistical analysis of age differences for continuous variables. The chi-square test was used to determine if differences in category variables were significant. The Shapiro–Wilk test was used to determine whether or not all metabolic values went against a normal distribution. Thus, the Kruskal–Wallis and Wilcoxon rank-sum tests, respectively, were used to assess the differences in lipid and glucose levels between and within groups. To look for correlations, Spearman’s correlation coefficient was employed. *p*-values of less than 0.05 were used to classify all variables as significant.

## 3. Results

**Figure 1:**
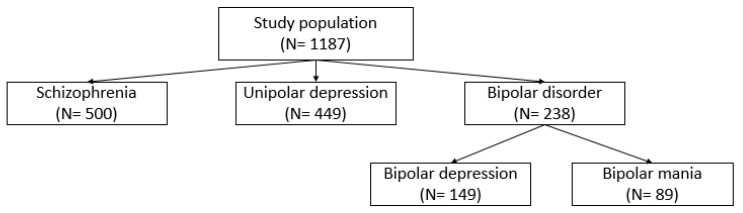
Stratification of patients.

**Figure 2:**
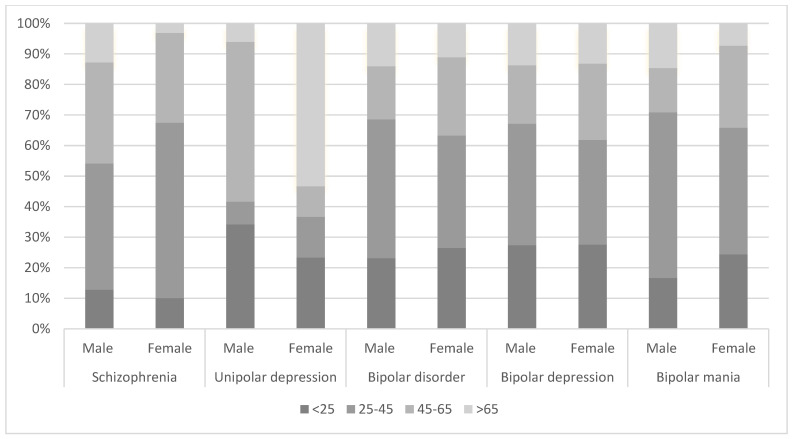
Age distribution for each mental disorder.

In this study, a total of 1187 patients were included in the analysis, and the majority were women (N = 675, 56.87%). Similarly, the majority of patients were women with schizophrenia (N = 258, 52.6%), unipolar depression (N = 300, 66.82%), and bipolar depression (N = 76, 51.01%); however, in bipolar disorder and bipolar mania, the majority of patients were men (N = 121, 50.84%; N = 48, 53.93%; respectively). The difference between gender groups, tested by the chi-square test was significant in women (X^2^ = 150.12, df = 2, *p* = 0.002), where bipolar mania had the lowest prevalence of women (N = 41, 46.07%). Figure 1 shows the stratification of patients into five diagnostic groups. Figure 2 shows the total number of patients with the age distribution within each diagnostic category.

The age (mean ± standard deviation) of the studied group was 43.09 ± 23.19. The subgroup 25–45 had the highest prevalence of 495 patients (34.74%). Schizophrenia had the highest prevalence among patients. The difference between age groups, tested by a one-way ANOVA test, was significant (F = 80.12, df = 2, *p* = 0.003). A Bonferroni test showed that only unipolar depression patients were significantly older than other disorders (*p* = 0.006). Table 1 shows the age and gender distribution of participants along with each mental disorder. 

This study revealed many significant correlations with age. There were weak significant correlations of LDL level with age in all the studied disorders (r = 0.31, *p* = 0.002) but no significant correlation in bipolar depression (*p* = 0.43); similarly, there were weak significant correlations of TC level with age in all the studied disorders (r = 0.35, *p* < 0.001) but no significance correlation in bipolar depression (*p* = 0.33).

In all the disorders, there was a very weak significant correlation (r = 0.01, *p* = 0.003) between HDL level and age; however, this correlation was not significant for unipolar depression (*p* = 0.59), bipolar disorder (*p* = 0.59), bipolar depression (*p* = 0.38), or bipolar mania (*p* = 0.93). Likewise, for every illness under study, there was a very weak significant association (r = 0.18, *p* = 0.001) between TGA level and age; however, this link was not significant for bipolar depression (*p* = 0.25). Additionally, there was a very weak significant link (r = 0.15, *p* = 0.008) between FPF level and age in all of the illnesses that were evaluated, but not in bipolar depression (*p* = 0.19). 

**Figure 3:**
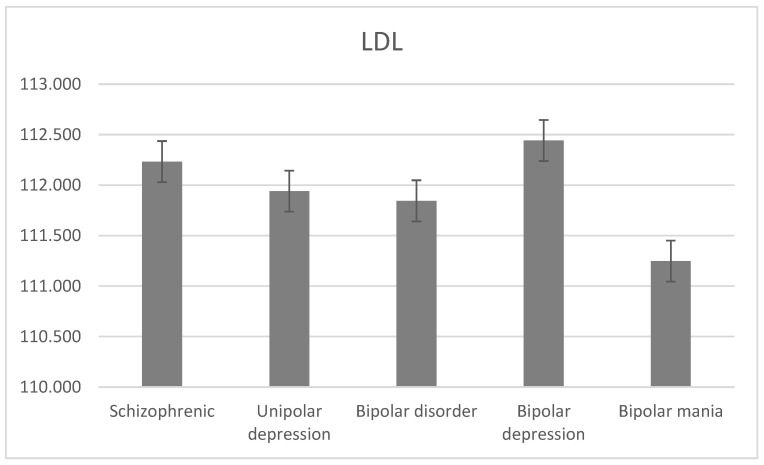
Mean LDL levels with standard error in different diagnostic groups.

The average level of LDL was 112.233 ± 51.222 mg/dL for schizophrenia, 111.940 ± 54.351 mg/dL for unipolar depression, 111.85 ± 34.66 mg/dL for bipolar disorder, 112.442 ± 36.178 mg/dL for bipolar depression, and 111.248 ± 33.140 mg/dL for bipolar mania. There was a statistically significant difference between the level of LDL and the diagnostic groups (H = 24.5, df = 2, *p* < 0.001; Figure 3). 

**Figure 4:**
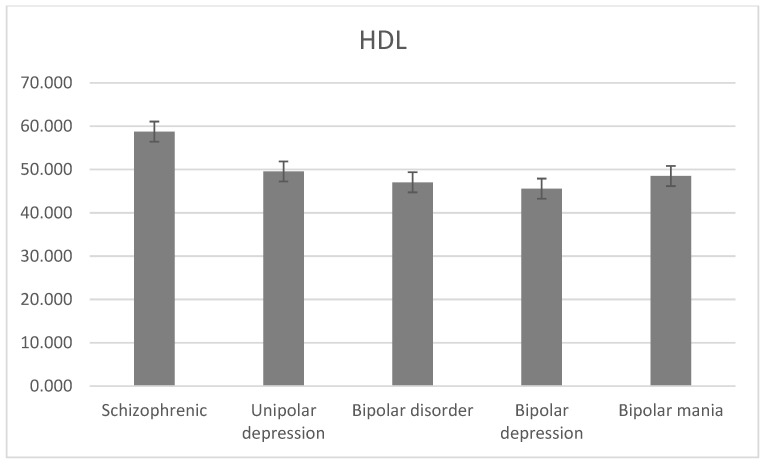
Mean HDL levels with standard error in different diagnostic groups.

The average level of HDL was 58.755 ± 16.198 mg/dL for schizophrenia, 49.554 ± 7.165 mg/dL for unipolar depression, 47.04 ± 12.472 mg/dL for bipolar disorder, 45.584 ± 12.128 mg/dL for bipolar depression, and 48.492 ± 12.816 mg/dL for bipolar mania. There was a statistically significant difference between the level of HDL and the diagnostic groups (H = 19.7, df = 3, *p* < 0.001; Figure 4).

**Figure 5:**
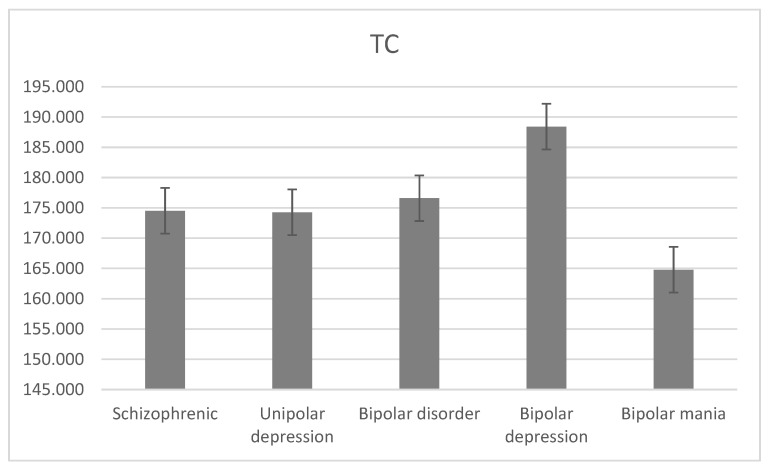
Mean TC levels with standard error in different diagnostic groups.

The average level of TC was 174.514 ± 25.858 mg/dL for schizophrenia, 174.263 ± 29.103 mg/dL for unipolar depression, 176.597 ± 38.942 mg/dL for bipolar disorder, 188.403 ± 37.396 mg/dL for bipolar depression, and 164.790 ± 40.488 mg/dL for bipolar mania. There was a statistically significant difference between the level of TC and the diagnostic groups (H = 25.9, df = 3, *p* < 0.001; Figure 5).

**Figure 6:**
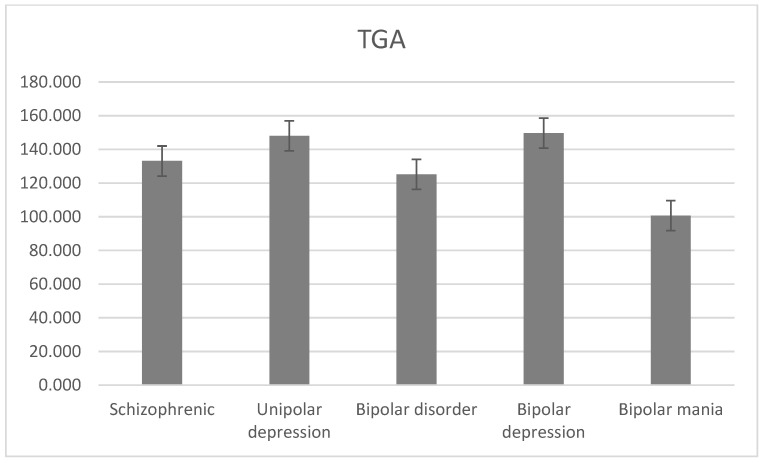
Mean TGA levels with standard error in different diagnostic groups.

The average level of TGA was 133.102 ± 34.451 mg/dL for schizophrenia, 148.015 ± 34.018 mg/dL for unipolar depression, 125.182 ± 75.644 mg/dL for bipolar disorder, 149.685 ± 96.951 mg/dL for bipolar depression, and 100.679 ± 54.337 mg/dL for bipolar mania. There was a statistically significant difference between the level of TGA and the diagnostic groups (H = 21.1, df = 2, *p* < 0.001; Figure 6).

**Figure 7:**
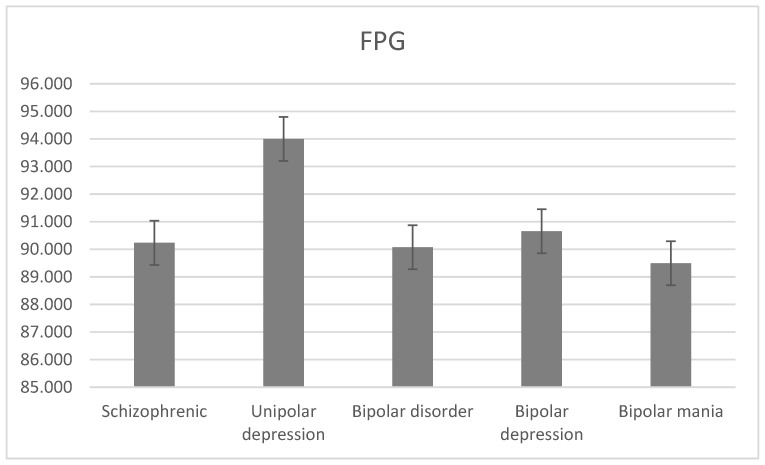
Mean FPG levels with standard error in different diagnostic groups.

The average level of FPG was 90.232 ± 20.833 mg/dL for schizophrenia, 94.000 ± 21.453 mg/dL for unipolar depression, 90.07 ± 16.342 mg/dL for bipolar disorder, 90.654 ± 17.984 mg/dL for bipolar depression, and 89.492 ± 14.700 mg/dL for bipolar mania. There was a statistically significant difference between the level of FPG and the diagnostic groups (H = 21.1, df = 2, *p* < 0.001; Figure 7).

The differences in glucose and lipid levels between the sex groups were analyzed. The sex distribution in diagnosis and odds ratios (ORs), with schizophrenia as a reference, of patients having high LDL, low HDL, high TC, high TGA, and high FPG for each psychiatric disorder along with gender are shown in Table 2.

The differences in glucose and lipid levels between the age groups were also analyzed. The age distribution in the diagnosis of LDL, HDL, TC, TGA, and FPG for each psychiatric disorder along with four age groups (<25, 25–45, 45–65, and >65) is shown in Table 3.

Between the low HDL (*p* = 0.41) and high FPG (*p* = 0.61) diagnostic groups, there was no statistically significant difference. The diagnostic groupings of high LDL, high TC, and high TGA categories did, however, vary statistically significantly (*p* < 0.001). When individuals with bipolar mania and depression were merged into a single bipolar disorder group, there was also a noteworthy distinction between the three groups. In comparison to the total mean age, patients with lower HDL and higher LDL, TC, TGA, and FPG levels were significantly older (*p* < 0.001).

## 4. Discussion

This prospective study evaluated the distribution of data outside of normal ranges and the levels of TGA, TC, HDL, LDL, and FPG in age subgroups of diagnostic categories in order to investigate variations in lipid and glucose levels in the acute stage of various psychiatric disorders.

The average LDL level was predominantly high in the bipolar depression group, where the differences between diagnostic group levels were statistically significant. For the schizophrenia group, the mean LDL level was the lowest (112.23 mg/dL) compared to two other studies, [6] and [7] (115.4 mg/dL and 139.9 mg/dL, respectively). There was a high risk of LDL levels surpassing the upper normal limit in both bipolar depression and unipolar depression groups, similar to [6]. Also, high LDL level prevalence was higher in women than men, where it was the lowest in schizophrenia (24.81%) and the highest in bipolar depression (38.16%), similar to [6]. There was a significant difference between men and women in LDL levels only for schizophrenia across all gender subgroups and certain age categories, contrary to [6]. Also, there was a significant difference in LDL levels between age groups for all disorders, except for the men group with bipolar depression and bipolar mania and the women group with unipolar depression. The patients aged between 40 and 60 years exhibited the highest levels of LDL levels for schizophrenia, unipolar depression, bipolar disorder, and bipolar mania, and those aged > 65 years exhibited the highest levels of LDL levels for bipolar depression. However, patients aged < 25 years showed the lowest levels of LDL levels for schizophrenia, unipolar depression, bipolar disorder, and bipolar mania, and those aged 25–45 years exhibited the lowest levels of LDL levels for bipolar depression. The average LDL level for unipolar depression patients (111.94 mg/dL) was approximately equal to [8] (113.3–117.9 mg/dL) and lower than [6] (125.7 mg/dL).

The average HDL level was mostly high in the schizophrenia group, where the differences between diagnostic group levels were not statistically significant. For the schizophrenia group, the mean HDL level was highest (58.76 mg/dL) compared to four other studies [6,7,9,10] (45.3 mg/Dl, 49.3 mg/dL, 43.7 mg/dL, and 51.1 mg/dL, respectively). The prevalence of low HDL was the highest (65.77%) among men with unipolar depression compared to the other lipid categories, which is in contrast to the results of [6], which showed that the prevalence was 59.70% among men with bipolar depression. Whereas it was highest in unipolar depression (63.70%) and lowest in bipolar mania (50.56%), low HDL levels were more common in women than in males. Additionally, 59.00% of the schizophrenia group had low HDL, a prevalence that was greater than the 29.80% and 52.80% of [6] and [11], respectively. 

There was a significant difference in HDL levels between men and women for all examined illnesses across most gender subgroups and most age categories, contrary to [6]. Nonetheless, for every clinical condition under investigation, there was no discernible variation in severity among age groups. In patients with unipolar depression, the average HDL level was 49.55 mg/dl, which is somewhat comparable to the findings of [6] and lower than the findings of [8] (52.0–53.0 mg/dL). A total of 63.70% of people had low HDL, which is three times greater than the research in [12] (21.70%) and higher than the 52.30% found in [6]. The prevalence of low HDL was also the same for individuals with bipolar depression (59.24% vs. 58.30%) and bipolar mania (50.56% vs. 50.60%) compared to [13].

The average level of TC was mostly high in the bipolar depression group compared to other groups. For the schizophrenia group, the average TC level was 174.51 mg/dL, which was lower than [6,7,9,10], which were 188.5 mg/dL, 204.9 mg/dL, 204.3 mg/dL, and 185.0 mg/dL, respectively. High TC level prevalence was higher in women than men, where it was the lowest in bipolar mania (16.85%) and the highest in bipolar depression (44.30%), similar to [6]. There was a statistically significant difference between men and women in TC levels in schizophrenia, unipolar depression, and bipolar mania across all gender subgroups and most age categories, similar to [6]. There was a significant difference between age categories for schizophrenia and bipolar disorder subgroups and bipolar depression and bipolar mania for the men group. Groups aged 25–45 and 45–65 had the highest levels of TC for all psychiatric disorders, and the lowest was in the youngest patients.

The average TGA level was mostly high in the bipolar depression group compared to other groups. For the schizophrenia group, the mean TGA level was the lowest (133.10 mg/dL) compared to four other studies [6,7,10,11], which were 139.8 mg/dL, 192.4 mg/dL, 159.1 mg/dL, and 162.6 mg/dL. Also, for the schizophrenia group, the high TGA level was higher (33.20%) compared to 32.80% in [6] and lower compared to 42.30% in [12]. Similarly, for the unipolar depression group, the prevalence of TGA was 39.09%, higher than both [6] and [13], which were 33.30% and 25.50%, respectively, and approximately equal to [14], which was 40.90%. Also, the prevalence of high TGA levels was higher in men than women, where it was the lowest in bipolar mania (21.57%) and the highest in bipolar depression (44.97%). Also, for the bipolar mania group, the prevalence of TGA was 21.57%, approximately equal to [6] and two times lower than [14] at values of 20.80% and 11.20%, respectively. There was a significant difference in TGA levels between men and women only in schizophrenia across all gender subgroups and most age categories, contrary to [6]. There were significant differences between age categories for schizophrenia and bipolar disorder subgroups. Groups aged 25–45 and 45–65 had the highest levels of TGA for all psychiatric disorders, where those in the <25 group had the lowest TGA levels, except for bipolar depression and bipolar mania; those in the >60 group had the highest TGA levels, and those in the 45–65 and 25–45 groups had the lowest level, respectively. The average TGA level for the unipolar depression group was 148.02, lower than both [6] and [8], which had values of 125.3 mg/dL and 117.0–153.0 mg/dL, respectively. Also, TGA was 159.85 and 142.14 for men and women, lower than both [6] and [15], which had values of 140.8 and 120.5 mg/dL, and 114.5 and 104.1 mg/dL, respectively.

The average FPG level was mostly high in the bipolar disorder group compared to other groups. For the schizophrenia group, the average FPG level was 90.23 mg/dL, lower than [6,10], which had values of 95.8 mg/dL and 94.8 mg/dL, respectively. Moreover, for the schizophrenia group, the prevalence of high FPG was 26.00%, slightly higher than [12] at 25.30% and lower than [6] at 29.70%. 

High FPG level prevalence was higher in women than men in all studied groups, where it was the highest in unipolar depression (36.08%) and the lowest in bipolar mania (23.48%), contrary to [6]. The FPG level was not significantly different between diagnostic groups; however, it was significantly different in certain age categories, namely, schizophrenia, unipolar depression, and bipolar depression. There were significant differences between age categories for all groups, except for the men and women subgroup for bipolar depression and bipolar mania, respectively. Groups aged 45–46 and >65 had the highest FPG level and younger patients had the lowest for all studied disorders, except for bipolar disorder, which had the highest level in the youngest group and lowest in the 25–45 age group. The average FPG level was 94.00 mg/dL, approximately equal to 94.8 mg/dL in [6] and lower than 96.00–98.00 mg/dL in [8]. The mean FPG level was 94.42 mg/dL for men and 93.79 mg/dL for women, higher than both [6] and [15]. 

Generally, it was noticed that lipid and glucose levels were correlated positively with age. This aligned with both [6] and [15], which stated that high metabolic syndrome was more common in the elderly population. Also, due to their increased vulnerability to metabolic problems, both women and older patients may account for a substantial portion of the reported outcomes.

This study sample’s non-population-based design captured the connections between mental diseases and metabolic dysfunctions better. A study reported differences with a large population may be explained in part by this [6]. 

Although this study did not differentiate between smoking and non-smoking patients, previous studies showed that smokers exhibited a greater frequency of metabolic syndrome and had distinct factors contributing to metabolic syndrome than non-smokers [16,17]. Also, this study did not differentiate between medicated and unmedicated patients. Previous studies showed that there was a correlation between metabolic parameters and medication prescribed. Chronic patients treated with antipsychotic medications have been shown to affect metabolic parameters [18].

## 5. Conclusions

Our results confirmed that low levels of HDL and high levels of triglycerides, cholesterol, LDL, and glucose were present in people with schizophrenia and mood disorders, both unipolar and bipolar. However, as we have demonstrated, there are differences in the nature and occurrence of these metabolic dysfunctions across different diagnostic categories. The incredibly high prevalence of various metabolic illnesses underlined the need to routinely monitor cholesterol and glucose levels in all patients. Women and patients over 45 years of age need extra attention since they are more fragile.

An important limitation of the work is that this study did not address the correlations between metabolic parameters with smoking and prescribed medication. However, this study’s strengths are its sizable sample size and capacity to compare three important clinical groups: bipolar illness, unipolar depression, and schizophrenia. Moreover, to our knowledge, there has been no previous investigation of the link between mental health illness and metabolic dysregulation among Jordanians. Participants were gathered from outpatient mental health clinics; as a result, this cohort was probably made up of people who were somewhat driven and could be more concerned with their physical and mental health than people who declined to be part of this study. Therefore, this cohort’s physical condition may not be typical of all mental outpatients in Jordan. This cohort may be even more burdened with physical co-morbidity and cardiovascular risk since it was likely that the physical health of people who declined to participate in physical health screening trials was not as regularly maintained.

## Figures and Tables

**Table 1:** Demographic characteristics of patients.

Diagnosis	Gender	Age Category				Total
		<25	25–45	45–65	>65	N (%)
Schizophrenia	Male	31	100	80	31	242 (48.4%)
	Female	26	148	76	8	258 (52.6%)
	Total N (%)	57 (11.4%)	248 (49.6%)	156 (31.2%)	39 (7.8%)	500 (100%)
Unipolar depression	Male	51	11	78	9	149 (33.18%)
	Female	70	40	30	160	300 (66.82%)
	Total N	121 (26.95%)	51 (11.36%)	108 (24.05%)	169 (37.64%)	449 (100%)
Bipolar disorder	Male	28	55	21	17	121 (50.84%)
	Female	31	43	30	13	117 (49.16%)
	Total N	59 (24.79%)	98 (41.18%)	51 (21.43%)	30 (12.61%)	238 (100%)
Bipolar depression	Male	20	29	14	10	73 (48.99%)
	Female	21	26	19	10	76 (51.01%)
	Total N	41 (27.52%)	55 (36.91%)	33 (22.15%)	20 (13.42%)	149 (100%)
Bipolar mania	Male	8	26	7	7	48 (53.93%)
	Female	10	17	11	3	41 (46.07%)
	Total N	18 (20.22%)	43 (48.31%)	18 (20.22%)	10 (20.22%)	89 (100%)
Total		237 (19.67%)	397 (33.45%)	315 (26.54%)	238 (20.05%)	1187 (100%)

**Table 2:** Sex distribution in the diagnostic groups and/or for abnormal ranges of lipids and glucose.

	High LDL N (%)	Low HDL N (%)	High TC N (%)	High TGA N (%)	High FPG N (%)
Schizophrenia OR	1.00	1.00	1.00	1.00	1.00
Men	72 (29.75%)	135 (55.79%)	61 (25.21%)	97 (40.08%)	49 (20.25%)
Women	64 (24.81%)	160 (62.02%)	83 (32.17%)	69 (26.74%)	81 (31.40%)
Schizophrenia	136 (27.20%)	295 (59.00%)	144 (28.80%)	166 (33.20%)	130 (26.00%)
Unipolar depression OR	1.79 [1.09–2.15]	0.78 [0.56–1.25]	2 [1.41–2.36]	1 [0.49–0.91]	1 [0.84–1.29]
Men	54 (36.24%)	98 (65.77%)	49 (32.89%)	69 (46.31%)	46 (30.87%)
Women	104 (34.67%)	188 (62.67%)	147 (49.00%)	107 (35.50%)	116 (38.67%)
Unipolar depression	158 (35.189%)	286 (63.697%)	196 (43.65%)	176 (39.087%)	162 (36.08%)
Bipolar disorder OR	1.56 [0.79–1.79]	1.01 [0.68–1.59]	1 [0.96–1.76]	1 [0.93–1.82]	1 [0.79–1.53]
Men	37 (30.58%)	71 (58.68%)	40 (33.06%)	55 (45.45%)	29 (23.97%)
Women	35 (29.91%)	70 (59.83%)	53 (45.30%)	36 (30.77%)	34 (29.06%)
Bipolar disorder	72 (30.25%)	141 (59.24%)	93 (39.08%)	91 (38.24%)	63 (26.47%)
Bipolar depression OR	1.59 [1.13–2.08]	1.35 [0.91–1.61]	1 [1.1–1.64]	1 [1.08–1.74]	1 [0.79–1.27]
Men	22 (30.14%)	45 (61.64%)	30 (41.10%)	36 (49.32%)	19 (26.03%)
Women	29 (38.16%)	45 (59.21%)	36 (47.37%)	31 (40.79%)	27 (35.00%)
Bipolar depression	51 (34.23%)	90 (60.40%)	66 (44.30%)	67 (44.97%)	46 (30.60%)
Bipolar mania OR	0.74 [0.36–1.32]	0.86 [0.49–1.39]	1 [0.55–1.98]	1 [0.41–0.89]	1 [0.31–1.29]
Men	8 (16.67%)	22 (45.83%)	3 (6.25%)	11 (22.92%)	9 (18.54%)
Women	13 (31.71%)	23 (56.10%)	12 (29.27%)	8 (20.00%)	12 (29.27%)
Bipolar mania	21 (23.60%)	45 (50.56%)	15 (16.85%)	19 (21.57%)	21 (23.48%)

**Table 3:** Age distribution in the diagnostic groups of lipids and glucose.

	<25	25–45	45–65	>65	Total	H	*p*
LDL [mg/dL]							
Male	100.34 ± 59.05	108.45 ± 20.18	119.42 ± 15.80	100.89 ± 80.10	110.07 ± 31.39	33.91	<0.001
Female	97.54 ± 20.15	113.71 ± 90.81	121.94 ± 50.10	105.91 ± 30.51	114.26 ± 69.83	20.40	<0.001
Schizophrenic	99.06 ± 41.31	111.59 ± 62.33	120.65 ± 32.51	101.92 ± 69.93	112.23 ± 51.22	19.60	<0.001
Z	0.40	−2.30	−0.08	−2.91	−2.90		
P	0.90	0.04	0.80	0.03	0.01		
Men	87.25 ± 89.07	118.44 ± 58.18	125.42 ± 43.80	118.89 ± 89.10	111.45 ± 63.09	11.29	0.01
Women	97.80 ± 20.15	109.7 ± 91.17	124.07 ± 60.10	116.85 ± 50.89	112.19 ± 50.01	4.90	0.40
Unipolar depression	93.35 ± 49.20	111.66 ± 84.05	125.05 ± 48.33	116.96 ± 52.92	111.94 ± 54.35	9.10	0.05
Z	0.90	0.25	−0.03	0.30	−0.24		
P	0.99	0.90	0.89	0.92	0.92		
Men	104.40 ± 33.1	105.450 ± 36.3	116.00 ± 35.1	125.65 ± 43.15	111.85 ± 37.17	4.10	0.09
Women	109.45 ± 40.1	103.950 ± 17.6	119.05 ± 42.85	119.65 ± 33.35	112.057 ± 32.62	6.66	0.06
Bipolar disorder	107.18 ± 37.04	104.617 ± 27.51	118.13 ± 40.14	123.80 ± 40.5	111.845 ± 34.57	2.90	0.06
Z	−0.98	−1.98	0.30	−0.25	−20.70		
P	0.40	0.07	0.90	0.85	0.40		
Men	125.70 ± 46.10	96.30 ± 40.20	125.20 ± 52.20	130.10 ± 42.20	114.53 ± 44.39	3.10	0.40
Women	126.80 ± 43.80	90.10 ± 4.00	110.10 ± 35.10	129.60 ± 46.50	110.44 ± 28.36	8.01	0.05
Bipolar depression	126.26 ± 44.92	93.37 ± 23.09	116.51 ± 42.35	129.85 ± 44.06	112.44 ± 36.18	10.81	0.01
Z	0.15	0.25	0.10	0.28	−0.25		
P	0.90	0.91	0.89	0.85	0.79	7.70	0.06
Men	83.10 ± 20.10	114.60 ± 32.40	106.80 ± 18.00	121.20 ± 44.10	109.18 ± 29.96	1.81	0.70
Women	92.10 ± 36.40	117.80 ± 31.20	128.00 ± 50.60	109.70 ± 20.20	113.68 ± 36.87	6.70	0.08
Bipolar mania	88.10 ± 29.16	115.87 ± 31.93	119.76 ± 37.92	117.75 ± 36.93	111.25 ± 33.14		
Z	−0.90	−1.90	0.23	−0.32	−2.70		
P	0.40	0.07	0.82	0.70	0.40		
HDL [mg/dL]							
Male	48.23 ± 10.08	60.45 ± 9.50	65.92 ± 15.80	44.89 ± 8.50	58.70 ± 11.53	30.70	0.01
Female	53.91 ± 7.89	55.78 ± 8.19	67.94 ± 50.10	43.98 ± 10.51	58.81 ± 20.58	17.50	0.00
Schizophrenic	50.82 ± 9.08	57.66 ± 8.72	66.90 ± 32.51	44.70 ± 8.91	58.76 ± 16.20	4.10	0.40
Z	−1.50	−1.81	−6.10	−1.10	−11.30		
P	0.10	0.00	0.00	0.74	0.00		
Men	38.25 ± 9.81	47.64 ± 5.18	50.02 ± 3.87	63.99 ± 9.18	46.66 ± 6.32	0.91	0.91
Women	43.80 ± 12.15	55.79 ± 8.19	40.27 ± 6.18	54.95 ± 5.70	50.99 ± 7.59	0.55	0.81
unipolar depression	41.46 ± 11.16	54.03 ± 7.54	47.31 ± 4.51	55.43 ± 5.89	49.55 ± 7.17	1.85	0.51
Z	−1.80	−2.80	−2.90	−2.15	−6.10		
P	0.06	0.00	0.01	0.01	0.00		
Men	45.54 ± 6.84	40.9 ± 9.26	38.68 ± 18.75	44.17 ± 15.0	41.69 ± 11.61	2.50	0.50
Women	53.64 ± 7.68	55.04 ± 13.57	47.15 ± 18.42	53.53 ± 13.5	52.69 ± 13.25	2.91	0.80
Bipolar disorder	49.73 ± 7.29	46.88 ± 11.32	43.68 ± 18.53	48.48 ± 14.62	47.04 ± 12.47	2.13	0.60
Z	−1.45	−4.10	−3.80	−3.10	−6.10		
P	0.20	0.00	0.00	0.01	0.00		
Men	40.18 ± 9.56	40.89 ± 5.12	36.41 ± 19.10	40.15 ± 11.20	39.73 ± 9.85	1.30	0.94
Women	54.18 ± 10.20	50.98 ± 14.25	46.12 ± 20.14	55.19 ± 12.20	51.20 ± 14.33	1.56	0.67
Bipolar depression	47.35 ± 9.89	45.66 ± 9.44	42.00 ± 19.70	47.67 ± 11.63	45.58 ± 12.13	2.15	0.65
Z	1.84	3.50	3.40	2.10	4.99		
P	0.06	0.00	0.01	0.01	<0.001	3.19	0.49
Men	50.89 ± 4.12	40.90 ± 13.40	40.95 ± 18.40	48.19 ± 18.80	43.64 ± 13.37	5.16	0.56
Women	53.10 ± 5.16	59.10 ± 12.89	48.18 ± 16.70	51.87 ± 14.80	54.18 ± 12.17	20.15	0.80
Bipolar mania	52.12 ± 4.70	48.10 ± 13.20	45.37 ± 17.36	49.29 ± 17.60	48.49 ± 12.82		
Z	1.50	2.19	3.46	0.98	2.01		
P	0.26	0.15	0.21	0.69	<0.001		
TC [mg/dL]							
Male	150.21 ± 89.04	165.95 ± 20.50	189.00 ± 55.70	190.080 ± 44.560	174.64 ± 43.99	H = 46.90	<0.001
Female	165.00 ± 3.82	175.97 ± 18.89	171.94 ± 10.10	198.980 ± 15.140	174.39 ± 14.67	H = 21.60	0.00
Schizophrenic	156.96 ± 5 0.17	171.93 ± 19.54	180.69 ± 33.48	191.906 ± 38.525	174.51 ± 28.86	H = 28.09	<0.001
Z	0.05	−3.40	−0.81	−2.90	−4.50		
P	0.80	0.006	0.45	0.005	0.001		
Men	140.25 ± 9.80	168.64 ± 5.18	180.02 ± 3.78	190.99 ± 9.18	166.23 ± 6.27	9.80	0.02
Women	160.92 ± 44.15	170.00 ± 38.13	175.85 ± 60.18	188.35 ± 35.70	178.25 ± 40.44	8.90	0.07
unipolar depression	152.21 ± 29.67	169.71 ± 31.02	178.86 ± 19.45	188.49 ± 34.29	174.26 ± 29.10	2.60	0.70
Z	−1.10	−1.80	0.50	−0.71	−2.90		
P	0.33	0.07	0.89	0.80	0.01		
Men	159.20 ± 21.18	173.20 ± 57.75	189.80 ± 42.9	189.60 ± 37.57	175.16 ± 44.08	40.78	<0.001
Women	167.95	174.80 ± 31.77	189.85 ± 41.60	194.55 ± 53.35	178.31 ± 33.70	10.50	<0.001
Bipolar disorder	164.1	173.75 ± 46.44	189.81 ± 42.11	191.01 ± 42.88	176.6 ± 38.94	24.60	<0.001
Z	−1.90	−2.50	−0.77	0.81	−1.81		
P	0.17	0.18	0.60	0.40	0.1		
Men	177.30 ± 17.25	190.80 ± 60.30	199.40 ± 50.20	190.10 ± 42.10	188.65 ± 44.08	3.08	0.40
Women	175.70 ± 1.80	188.70 ± 33.40	200.60 ± 50.10	189.30 ± 50.80	188.16 ± 31.13	8.95	0.01
Bipolar depression	176.48 ± 9.34	189.81 ± 47.58	200.09 ± 50.14	189.70 ± 45.87	188.40 ± 37.40	12.80	0.01
Z	−0.35	−0.50	−0.80	−0.34	−1.40		
P	0.70	0.71	0.55	0.73	0.20		
Men	141.10 ± 25.10	155.60 ± 55.20	180.20 ± 35.60	189.10 ± 33.03	161.66 ± 44.09	9.10	2.13
Women	160.20 ± 44.30	160.90 ± 30.13	179.10 ± 33.10	199.80 ± 55.90	168.46 ± 36.27	0.03	0.60
Bipolar mania	151.71 ± 35.77	157.70 ± 45.29	179.53 ± 34.07	192.31 ± 39.89	164.79 ± 40.49	80.55	0.02
Z	−1.10	−1.80	0.33	−0.07	−2.30		
P	0.28	0.05	0.80	0.99	0.02		
TGA [mg/dL]							
Male	110.21 ± 45.09	142.690 ± 29.57	146.90 ± 59.78	109.81 ± 38.98	135.71 ± 42.75	25.91	<0.001
Female	102.01 ± 20.12	136.04 ± 25.89	130.99 ± 30.89	120.98 ± 22.18	130.66 ± 26.67	20.15	<0.001
Schizophrenic	106.47 ± 33.70	138.72 ± 27.37	139.15 ± 45.71	112.10 ± 35.53	133.10 ± 34.45	23.10	<0.001
Z	2.03	5.90	1.80	−1.60	4.90		
P	0.035	<0.001	0.005	0.40	<0.001		
Men	140.25 ± 9.81	168.64 ± 5.18	170.02 ± 3.87	171.99 ± 9.18	159.85 ± 6.32	7.17	0.07
Women	136.42 ± 54.19	137.12 ± 58.18	142.91 ± 80.98	145.75 ± 36.14	142.14 ± 47.77	8.51	0.94
unipolar depression	138.03 ± 35.48	143.92 ± 46.75	162.49 ± 25.29	147.15 ± 34.70	148.02 ± 34.02	11.59	0.04
Z	1.67	0.60	0.31	1.3	1.20		
P	0.11	0.58	0.80	0.26	0.29		
Men	153.85 ±103.0	134.65 ± 116.1	145.45 ± 93.9	160.85 ± 95.25	140.21 ± 99.42	24.70	<0.001
Women	127.95 ± 63.6	82.90 ± 33.75	116.20 ± 56.9	140.50 ± 66.7	110.34 ± 51.21	11.40	0.01
Bipolar disorder	139.63 ± 81.36	109.6 ± 77.54	127.88 ± 71.37	153.93 ± 85.67	125.18 ± 75.64	27.46	<0.001
Z	0.01	0.1	0.40	1.41	0.60		
P	0.89	0.81	0.82	0.09	0.70		
Men	163.50 ± 88.10	199.20 ± 197.10	145.70 ± 75.30	175.10 ± 100.30	175.86 ± 130.62	0.75	0.86
Women	155.60 ± 76.60	80.50 ± 41.90	128.20 ± 70.60	166.90 ± 85.10	124.54 ± 64.35	10.77	0.16
Bipolar depression	159.45 ± 82.21	143.09 ± 123.73	135.62 ± 72.59	171.00 ± 93.71	149.68 ± 96.95	7.66	0.07
Z	1.73	0.60	0.31	0.20	1.08		
P	0.12	0.55	0.80	0.23	0.30		
Men	144.20 ± 117.90	70.10 ± 35.10	145.20 ± 112.50	146.60 ± 90.20	104.56 ± 68.22	5.55	0.08
Women	100.30 ± 50.60	85.30 ± 25.60	104.20 ± 43.20	114.10 ± 48.30	96.14 ± 38.08	0.90	0.85
Bipolar mania	119.81 ± 80.51	76.11 ± 31.34	120.14 ± 70.15	136.85 ± 77.63	100.68 ± 54.34	7.45	0.07
Z	−1.85	−0.50	0.51	1.11	−0.70		
P	0.08	0.60	0.60	0.29	0.53		
FPG [mg/dL]							
Male	99.30 ± 42.30	88.90 ± 15.10	99.30 ± 22.90	93.30 ± 20.30	94.23 ± 21.83	25.30	<0.001
Female	8.13 ± 16.10	93.70 ± 17.00	97.10 ± 23.60	106.60 ± 50.70	86.48 ± 19.90	22.37	<0.001
Schizophrenic	57.71 ± 30.35	91.76 ± 16.23	98.23 ± 23.24	96.03 ± 26.54	90.23 ± 20.83	37.00	<0.001
Z	0.80	1.80	2.02	−1.65	0.90		
P	0.33	0.06	0.03	0.08	0.40		
Men	88.30 ± 10.20	83.30 ± 12.20	99.10 ± 33.20	102.20 ± 35.60	94.42 ± 23.92	23.50	<0.001
Women	86.70 ± 10.90	84.60 ± 16.90	93.20 ± 16.70	99.30 ± 25.80	93.79 ± 20.23	35.70	<0.001
unipolar depression	87.37 ± 10.60	84.32 ± 15.89	97.46 ± 28.62	99.45 ± 26.32	94.00 ± 21.45	53.74	<0.001
Z	−0.70	0.85	1.03	2.30	1.70		
P	0.52	0.41	0.41	0.03	0.10		
Men	83.20 ± 10.05	85.95 ± 15.90	94.50 ± 17.45	99.56 ± 15.79	88.38 ± 14.55	9.03	0.02
Women	82.45 ± 12.45	90.15 ± 7.1	103.60 ± 40.5	93.82 ± 18.95	92.06 ± 18.35	23.70	<0.001
Bipolar disorder	82.92 ± 11.38	87.55 ± 11.99	99.87 ± 31.13	97.54 ± 17.08	90.07 ± 16.34	27.13	<0.001
Z	0.02	−0.18	−2.10	1.33	−0.90		
P	0.98	0.50	0.05	0.30	0.45		
Men	85.70 ± 10.80	91.20 ± 20.50	90.60 ± 17.30	99.01 ± 17.07	90.65 ± 16.76	6.70	0.08
Women	79.30 ± 10.90	89.70 ± 9.30	101.70 ± 40.50	96.04 ± 22.30	90.66 ± 19.25	8.71	0.04
Bipolar depression	82.42 ± 10.85	90.49 ± 15.21	96.99 ± 30.66	97.53 ± 19.34	90.65 ± 17.98	10.55	0.02
Z	−1.70	1.70	−2.25	−0.25	−1.71		
P	0.12	0.35	0.03	0.08	0.11		
Men	80.70 ± 9.30	80.70 ± 11.30	98.40 ± 17.60	100.10 ± 14.50	86.11 ± 12.35	8.29	0.03
Women	85.60 ± 14.00	90.60 ± 4.89	105.50 ± 40.50	91.60 ± 15.60	93.45 ± 17.45	5.20	0.17
Bipolar mania	83.42 ± 11.91	84.61 ± 8.77	102.74 ± 31.59	97.55 ± 14.83	89.49 ± 14.70	13.90	0.00
Z	0.55	−1.30	−0.52	1.31	−1.12		
P	0.61	0.40	0.71	0.14	0.28		

## Data Availability

Data are available upon reasonable request.

## References

[1] Wainberg M.L., Scorza P., Shultz J.M., Helpman L., Mootz J.J., Johnson K.A., Neria Y., Bradford J.-M.E., Oquendo M.A., Arbuckle M.R. (2017). Challenges and opportunities in global mental health: A research-to-practice perspective. Curr. Psychiatry Rep..

[2] Heiberg I.H., Jacobsen B.K., Balteskard L., Bramness J.G., Næss Ø., Ystrom E., Reichborn-Kjennerud T., Hultman C.M., Nesvåg R., Høye A. (2019). Undiagnosed cardiovascular disease prior to cardiovascular death in individuals with severe mental illness. Acta Psychiatr. Scand..

[3] World Health Organization (WHO) (2017). Depression and Other Common Mental Disorders: Global Health Estimates. https://www.who.int/publications-detail-redirect/depression-global-health-estimates.

[4] Wysokiński A., Strzelecki D., Kłoszewska I. (2015). Levels of triglycerides, cholesterol, LDL, HDL and glucose in patients with schizophrenia, unipolar depression and bipolar disorder. Diabetes Metab. Syndr. Clin. Res. Rev..

[5] Grundy S.M., Stone N.J., Bailey A.L., Beam C., Birtcher K.K., Blumenthal R.S., Braun L.T., de Ferranti S., Faiella-Tommasino J., Forman D.E. (2019). 2018 AHA/ACC/AACVPR/AAPA/ABC/ACPM/ADA/AGS/APhA/ASPC/NLA/PCNA guideline on the management of blood cholesterol: Executive summary: A report of the American College of Cardiology/American Heart Association Task Force on Clinical Practice Guidelines. J. Am. Coll. Cardiol..

[6] Alexander C.M., Landsman P.B., Teutsch S.M., Haffner S.M. (2003). NCEP-defined metabolic syndrome, diabetes, and prevalence of coronary heart disease among NHANES III participants age 50 years and older. Diabetes.

[7] Vaccarino V., McClure C., Johnson B.D., Sheps D.S., Bittner V., Rutledge T., Shaw L.J., Sopko G., Olson M.B., Krantz D.S. (2008). Depression, the metabolic syndrome and cardiovascular risk. Psychosom. Med..

[8] Goff D.C., Sullivan L.M., McEvoy J.P., Meyer J.M., Nasrallah H.A., Daumit G.L., Lamberti S., D’Agostino R.B., Stroup T.S., Davis S. (2005). A comparison of ten-year cardiac risk estimates in schizophrenia patients from the CATIE study and matched controls. Schizophr. Res..

[9] de Leon J., Susce M.T., Johnson M., Hardin M., Pointer L., Ruaño G., Windemuth A., Diaz F.J. (2007). A clinical study of the association of antipsychotics with hyperlipidemia. Schizophr. Res..

[10] Wysokiński A., Kowman M., Kłoszewska I. (2012). The prevalence of metabolic syndrome and Framingham cardiovascular risk scores in adult inpatients taking antipsychotics-a retrospective medical records review. Psychiatr. Danub..

[11] De Hert M.A., van Winkel R., Van Eyck D., Hanssens L., Wampers M., Scheen A., Peuskens J. (2006). Prevalence of the metabolic syndrome in patients with schizophrenia treated with antipsychotic medication. Schizophr. Res..

[12] Richter N., Juckel G., Assion H.-J. (2010). Metabolic syndrome: A follow-up study of acute depressive inpatients. Eur. Arch. Psychiatry Clin. Neurosci..

[13] Sicras-Mainar A., Blanca-Tamayo M., Rejas-Gutiérrez J., Navarro-Artieda R. (2008). Metabolic syndrome in outpatients receiving antipsychotic therapy in routine clinical practice: A cross-sectional assessment of a primary health care database. Eur. Psychiatry.

[14] Kinder L.S., Carnethon M.R., Palaniappan L.P., King A.C., Fortmann S.P. (2004). Depression and the metabolic syndrome in young adults: Findings from the third national health and nutrition examination survey. Psychosom. Med..

[15] Mozaffarian D., Kamineni A., Prineas R.J., Siscovick D.S. (2008). Metabolic syndrome and mortality in older adults: The Cardiovascular Health Study. Arch. Intern. Med..

[16] Nadalin S., Peitl V., Karlović D., Huskić S., Zatković L., Buretić-Tomljanović A. (2023). Correlations Between Clinical and Metabolic Variables and Smoking among Antipsychotic- Naïve First-Episode and Nonadherent Chronic Patients with Psychosis. Arch. Psychiatry Res. Int. J. Psychiatry Relat. Sci..

[17] Li Z., Wang S., Chen Y., Wu X., Gu Y., Lang X., Wu F., Zhang X.Y. (2021). Smoking affects the patterns of metabolic disorders and metabolic syndrome in patients with first-episode drug-naive schizophrenia: A large sample study based on the Chinese Han population. Int. J. Neuropsychopharmacol..

[18] Sonnenschein S.F. (2020). State-Dependent Dopamine System Regulation Using Current and Novel Antipsychotic Drug Mechanisms: Developmental Implications in a Schizophrenia Model. Ph.D. Thesis.

